# Increasing benthic vent formation: a threat to Japan’s ancient lake

**DOI:** 10.1038/s41598-021-83649-4

**Published:** 2021-02-18

**Authors:** Michio Kumagai, Richard D. Robarts, Yasuaki Aota

**Affiliations:** 1 Ritsumeikan University, Noji-Higashi 1-1-1, Kusatsu, Shiga 525-0058 Japan; 2 World Water and Climate Foundation, 1348 Crown Isle Blvd, Courtenay, B.C V9N 0E2 Canada

**Keywords:** Environmental sciences, Limnology, Natural hazards, Solid Earth sciences

## Abstract

An autonomous underwater vehicle (AUV) was deployed in Lake Biwa from 2000 to 2012. In December 2009, ebullition of turbid water was first found in the deepest area (> 90 m) of the North Basin. Follow-up investigations in April and December 2010 and January 2012 confirmed the existence of benthic vents similar to the vents observed in other deep lakes. Importantly, vent numbers per unit travel distance in Lake Biwa dramatically increased from only two vents (0.37 vents km^−1^) in December 2009 to 54 vents (5.28 vents km^−1^) in January 2012, which could be related to recent tectonic activity in Japan, e.g., the M9.1 Tohoku earthquake in March 2011 and slow earthquakes along the Nankai Trough from 2006 to 2018. Continuous back-up investigations from 2014 to 2019 revealed additional benthic vents in the same area. The sudden increase in benthic vent activity (liquid and gaseous ebullitions) have significant potential to alter lake biogeochemistry and, ultimately, degrade Japan’s major drinking water source and may be a harbinger of major crustal change in the near future.

## Introduction

### Benthic vents

Lake Biwa, the oldest and largest tectonic lake in Japan, formed almost 4.2 million years ago in an area 100 km south of its present location. Plate tectonic activity is believed to have moved the lake to the present location about 0.4–1.5 million years ago^1,2^. The lake volume is 27.5 km^3^, or about 34% of Japan’s surface fresh water^3^. Lake Biwa is the drinking water source for > 14 million people living in the Kinki District, including the areas of Osaka, Kobe, Kyoto, Nara and Shiga. The lake thus is a precious freshwater resource that requires close monitoring and strict conservation^3,4^.

**Figure 1:**
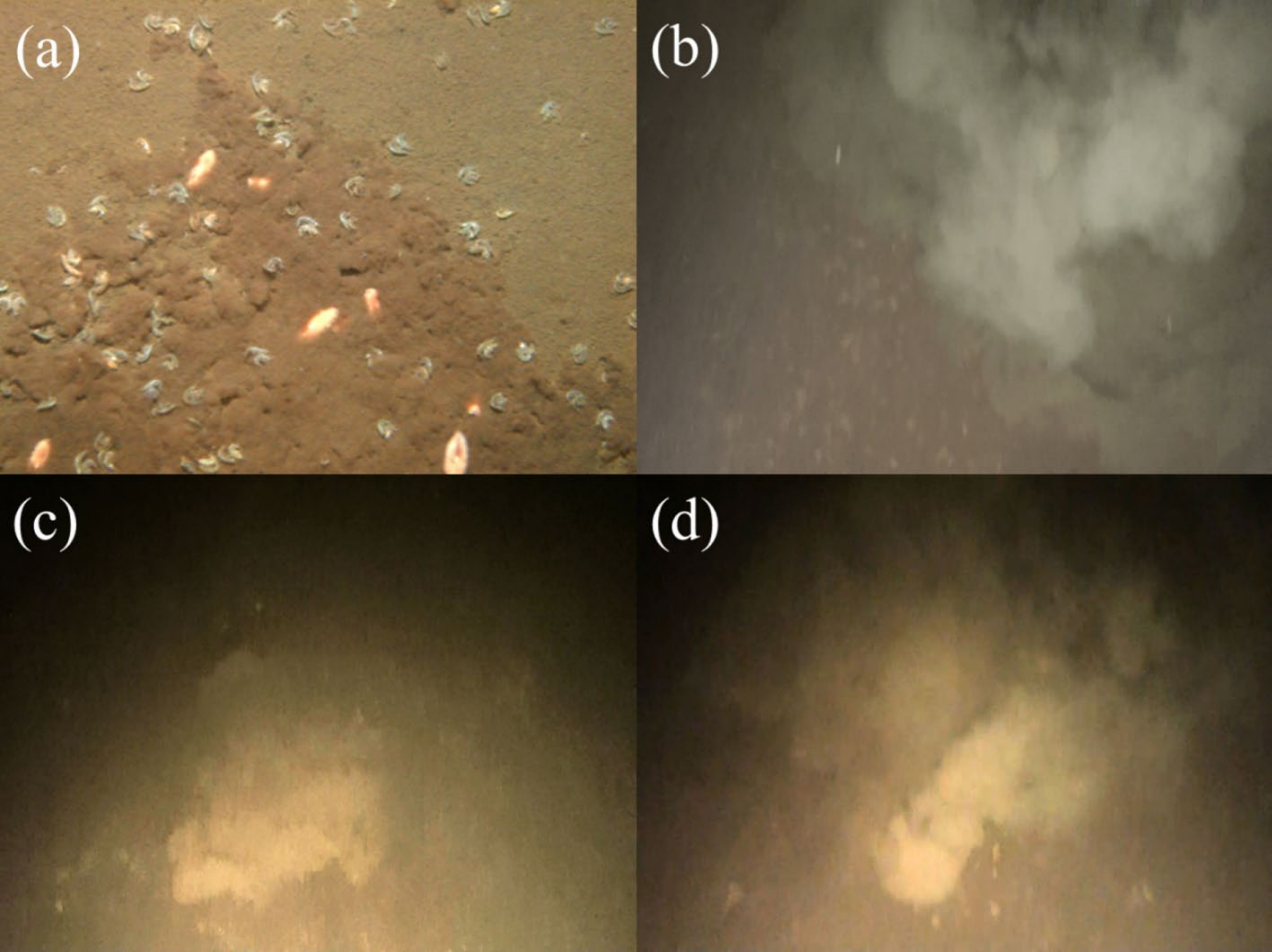
Photographs and images (from video film) taken by AUV “Tantan” in Lake Biwa at almost the same position as Fig. 2b. (**a**) Photo of benthic planarians (*Bdellocephala annandalei*), amphipods (*Jesogammarus annandalei*) and sediment disturbance near the deepest area on 25 August 2006. This digital camera image covers about 0.4 m in the horizontal and 0.3 m in the vertical planes. (**b**) A video image of a vent with high turbidity ebullition on 27 December 2009. The scale of this vent is nearly 2 m wide, 2 m deep and 1 m high. (**c**) Similar video image of a vent with a height of 0.5 m taken on 17 December 2010. (**d**) Video image of a vent detected with a height of 0.5 m and growing with a 1 m vertical scale and 4 m horizontal scale taken on 7 January 2012. The locations where images (**a**–**d**) were obtained are indicated in Fig. 3.

Using high-definition video and still pictures obtained with the AUV “Tantan”^5^, we documented interesting phenomena occurring on the lake bottom. In August 2006 (Fig. 1a), imagery from Tantan showed aggregations of benthic invertebrates that are endemic to the lake—specifically planarians (*Bdellocephala annandalei*)^6^ and amphipods (*Jesogammarus annandalei*)^7,8^—in a narrow 0.4 m × 0.3 m area. Evidence of initial sediment disturbance near the lake’s deepest site in the North Basin (613 km^2^, maximum depth 104.1 m) was also found. These results warranted additional surveys and analyses to better understand if the high biomass and localized concentration of benthic organisms in such a limited area is related to food availability, and most importantly, to get a better understanding of what is disturbing the lake sediments. Although in principle turbidity can be due to bioturbation^9^, given the magnitude of the turbulence required and the small animals we observed at this site, this seemed an unlikely explanation. This pronounced bottom ebullition was not apparent in any of the images recorded before 2006 and was the focus of the present study.

Tantan was deployed on 27 December 2009 in an area > 90 m deep in the North Basin of Lake Biwa. Its video cameras revealed a conspicuous vent (Fig. 1b) with vertical temperature instabilities and turbid water originating from natural sources. Similar environments have been reported in Lake Baikal with a deep-towed camera^10^, in Lake Tanganyika by scuba-diving investigation^11^ and Lake Taupo with a manned submersible vehicle^12^, where warm water ebullition from hydrothermal vents caused the formation of bacterial colonies. In Japan, some hydrothermal vents were observed in other lakes, such as Lake Mashu^13^ and Lake Towada^14^, and in marine areas such as Kagoshima Bay^15^. However, all of these are due to volcanic activities, whereas the Lake Biwa benthic vents are due to tectonic movement and further differ from them in their sudden appearance and increase over the last decades.

Observations in April 2010 were similar to those in December 2009, but the results in December 2010 and January 2012 showed a dramatic contrast: the benthic boundary layer was much less transparent due to turbid water (1.01 ± 0.11 FTU (Formazin Turbidity Unit), N = 9 in 2006; 2.19 ± 0.42 FTU, N = 19 in 2009; 2.63 ± 0.21 FTU, N = 6 in 2010; 3.23 ± 0.33 FTU, N = 5 in 2012), and many additional vents and plumes were detected along the observation lines (Table 1). The number of vents per unit Tantan travel distance was corroborated from two sets of high-definition video camera images. We carefully identified vents with sediment ebullitions (Fig. 1b–d) without any external disturbance, such as fish bioturbation. These data indicated there were no vents before December 2009, but there were 0.37 vents km^−1^ on 25–27 December 2009 and 5.28 vents km^−1^ on 5–8 January 2012 (Table 1).

**Table 1:** Number of vents detected by AUV “Tantan”, its cruising distance and unit distance density of vents from 2002 to 2012.

Date	Number of vents (N)	Distance (km)	Line density (N km^−1^)	SD (N km^−1^)
2002	0	21.73	0.00	ND
2003	0	17.10	0.00	ND
2004	0	18.87	0.00	ND
2005	0	16.81	0.00	ND
2006	0	30.11	0.00	ND
2007	0	45.61	0.00	ND
Dec 17–21 2008	0	11.72	0.00	ND
Nov 8–11 2009	0	11.83	0.00	ND
Dec 25–27 2009	2	5.43	0.37*	ND
Apr 26–28 2010	7	7.65	0.44**	0.39
Dec 16–18 2010	23	10.84	3.43**	1.16
Jan 5–8 2012	54	12.64	5.28**	3.76

*Indicates number of vents over distance.

**Evaluated from linear regression on number of vents against distance.

## Confirmation of vent activities

**Figure 2:**
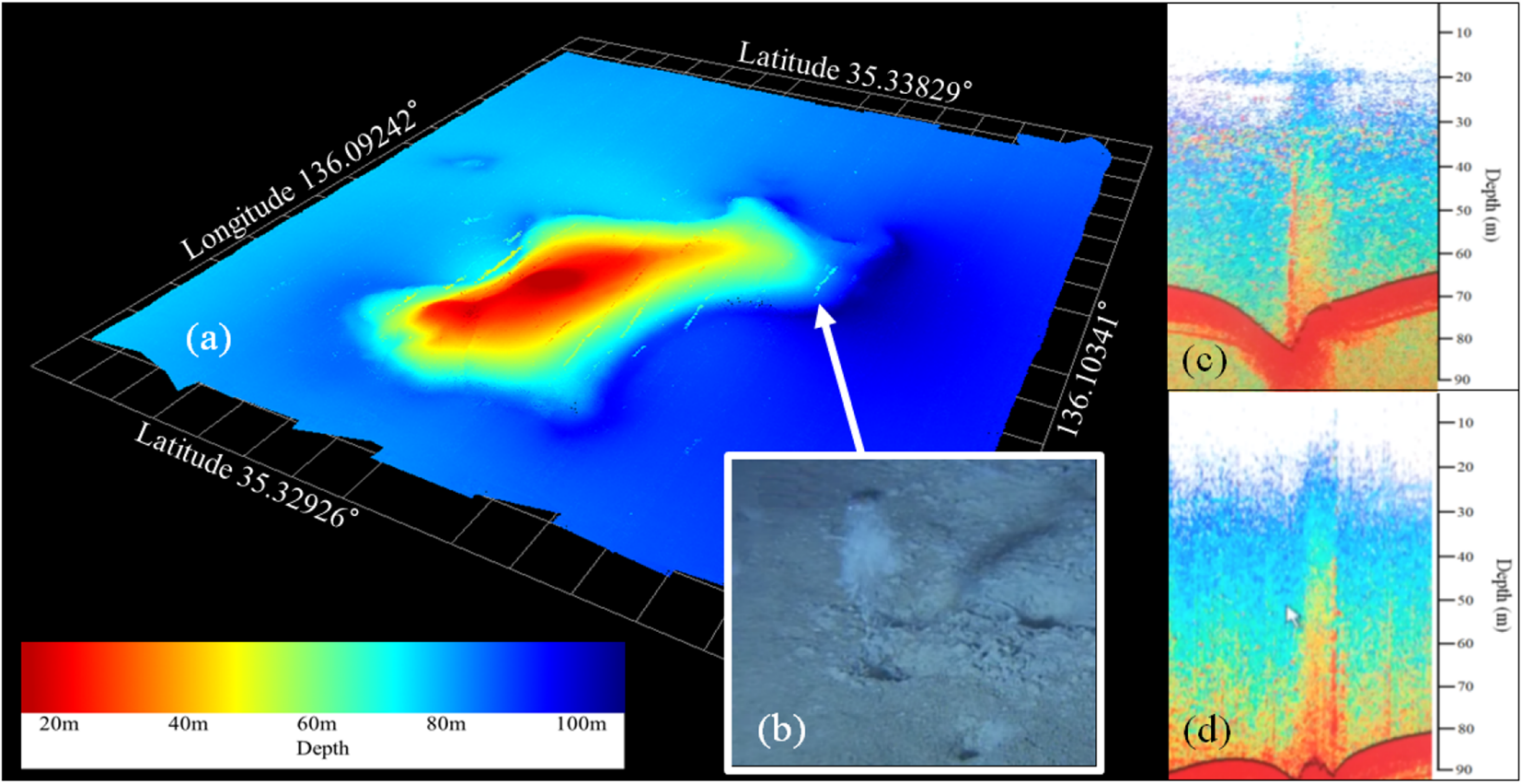
Schematic images of benthic vents in Lake Biwa. (**a**) The stereoscopic bottom image and gas bubble ebullitions measured by multi-beam sonar (MBS), where the dark red color indicates the shallow area (20 m depth) that used to be a submerged mountain peak and the dark blue color indicates the deepest area of Lake Biwa (104 m depth), (**b**) holes and intermittent gas bubbles observed by the ROV (NHK) with benthic vent location indicated by an arrow in the MBS image, and both (**c**) and (**d**) vertical plumes from a benthic vent observed by a quantitative echo sounder, where the vertical scale on the right is water depth. Gas bubbles formed vertical streaming lines with small bubble cluster segments, which may contribute to vertical material transport. Image (**c**) shows the horizontal spread of bubbles at the thermocline located at about 20 m, and in (**d**) the bubbles reached the water surface as no thermocline was present. The locations where images (**c**,**d**) were obtained are indicated in Fig. 3.

To confirm vent activity, we conducted additional surveys using a multi-beam echo sounder, a quantitative echo sounder, and a ROV from 2014 to 2018. A wide view of bottom topography was provided by the multi-beam echo sounder (Fig. 2a), and several gas ebullitions around the base of the submerged mound (dark red color) were found with vertical streaming lines of small clumped bubble segments. These gasses transport materials, such as nutrients, from the bottom sediment to overlying water (Fig. 2c,d), and may enhance water column microbial production. Indeed, average phytoplankton production in Lake Biwa increased from 574 mgC m^−2^ day^−1^ in 2006/2007 to 1104 mgC m^−2^ day^−1^ in 2017/18 ^16^.

ROV monitoring of benthic vents was near the deepest point of Lake Biwa on 15 May 2014. Several vents were found (Fig. 2b) where gas bubbles emerged intermittently at nearly 10 s intervals. Many large sized *Daphnia* species were observed around these vents, possibly *Daphnia pulicaria*, which is a zooplankton species that suddenly appeared after 1999 in the offshore zone of Lake Biwa^17^. We also found some microbial mats around the holes, which are typical features of hydrothermal vents in lakes Baikal^10^, Tanganyika^11^ and Taupo^12^. We collected two gas samples with the ROV and analyzed the gas bubbles emitted from the Lake Biwa holes. Methane was the major constituent (> 99%), which is known to support bacterial growth and could explain the large *Daphnia* spp. population that was observed^18,19^. Some dissolved methane concentrations in Lake Biwa were measured before^20,21^, but it is difficult to take gas samples without any special devices such as an ROV attached sampler or human occupied vehicle^22^.

Gas plumes (Fig. 2c) have also been observed at several sites using a quantitative echo sounder from 2012 to 2019 as further evidence of vent existence. As these gas plumes were identified with target strengths between − 60 dB and − 50 dB, the bubble size was estimated as ~ 40 mm in diameter^23^. The bubbles spread horizontally along the bottom of the thermocline in the stratified season (Fig. 2c) and reached the water surface during the mixing season (Fig. 2d)^24^. Such gas movement is known to contribute to vertical material transport, which can be another important mechanism of internal nutrient loading^25^, and can additionally contribute to atmospheric greenhouse gas production. Gas ebullition from the sediments of wet ecosystems is a GHG pathway that has long been underestimated but generally dominates emissions^26^.

## Alternative origins and locations of vents

To examine the possibility that the marked high turbidity near the bottom of Lake Biwa may be due in part to sediment re-suspension arising from shear instability in the benthic boundary layer, we deployed an acoustic Doppler current profiler (ADCP) to measure water velocity profiles every half an hour for 551 days from 2010 to 2012. Bottom shear stress was calculated from 31,898 field data profiles^27^. The results showed that only 29 profiles (21 in 2010, 1 in 2011 and 7 in 2012) were > 10 Nm^−2^, the critical bottom stress needed to resuspend bottom sediments^28^, while 96.3% (30,727 profiles) were < 1.0 Nm^−2^. The maximum value greater than critical shear stress was 19.95 Nm^−2^ measured in 2010. The probability of generating high bottom stress due to shear instability is < 0.1%, which clearly does not explain the increased and continuing turbidity from 2010 to 2012.

Sediment ebullition could also be the result of deep groundwater inflows. As Lake Biwa is surrounded by mountains, groundwater seepage can be found in many places, but these are mainly concentrated in areas < 40 m deep^29^ near the shore. The recent benthic vents, however, are unlikely to be caused by ordinary groundwater inflows because the recent rapid increase of lake turbidity and the ebullition locations in areas > 80 m deep do not coincide with the area of groundwater seepage previously observed^30,31^.

Thus, we conclude that neither groundwater inflows or bottom shear stresses were responsible for the sudden recent turbidity observed in the Tantan surveys. Rather, we believe that the evidence suggests that benthic vents are the cause, and when seeking possible mechanisms of their formation in Lake Biwa, the lake’s geologic history and formation^2,32^ becomes crucial.

## Geologic history of Lake Biwa

The present area of Lake Biwa is believed to have been mountainous terrain a few million years ago, but then subsided at about 0.74 mm year^−1^. This occurred because it lies on the northwest section of a crustal block that was affected by a change in direction of compression, first from south to north then from east to west, owing to complex plate tectonic interactions in the central island^2^.

**Figure 3:**
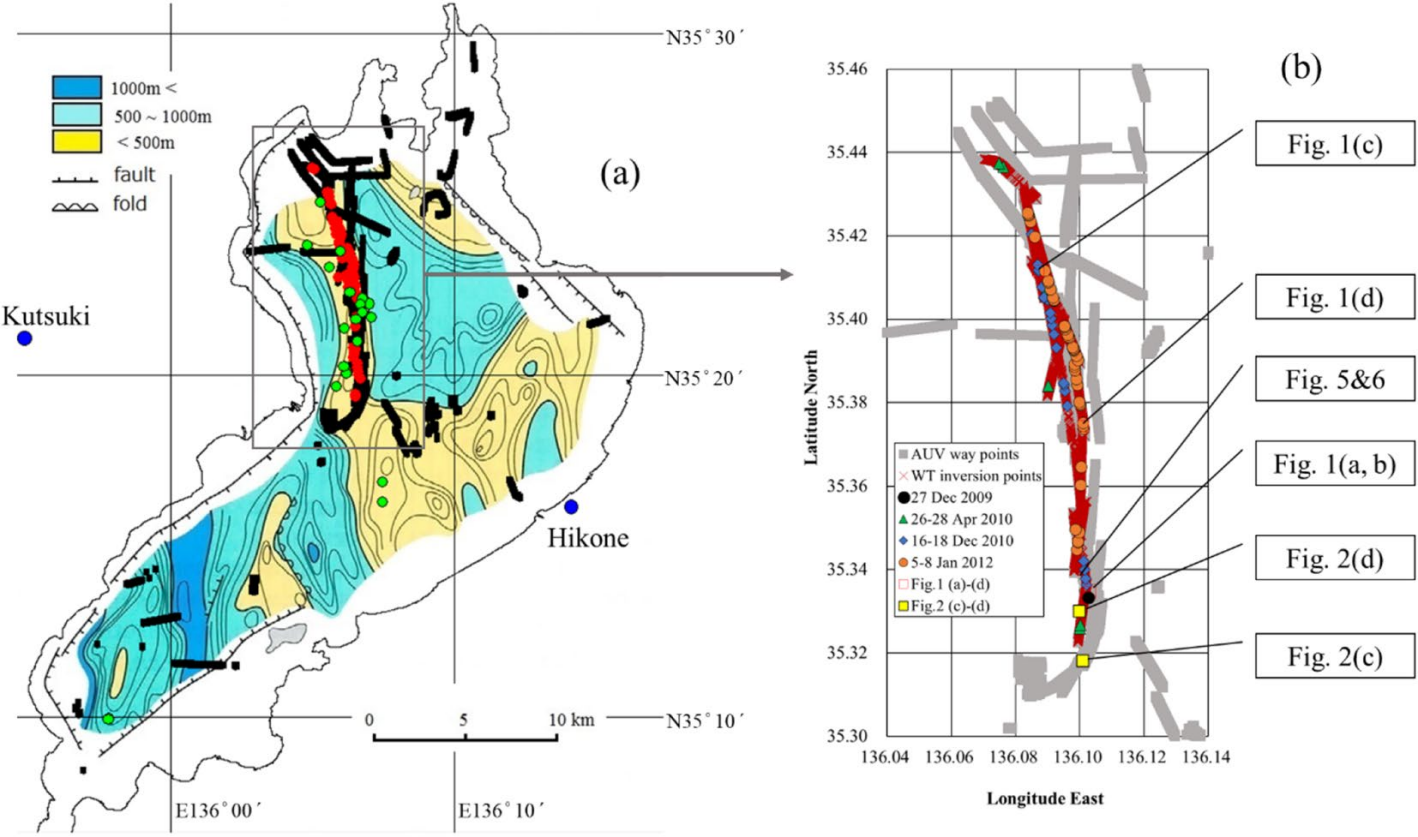
Waypoint locations for the AUV Tantan operated from 2001 to 2012 and the benthic vents detected from 2009 to 2012. (**a**) The way points (filled black rectangles) and the vents (orange circles) are plotted on the modified basement rock map of Lake Biwa with sediment thickness indicated with a color index (after Uemura and Taishi, 1990). The green circles indicate large ebullitions of gas and water observed using a quantitative echo sounder from 2012 to 2019. Noteworthy is that these vents are located along the shallow sedimentation region (< 500 m) indicated by light yellow, which may correspond to an ancient mountain range of several million years ago that gradually sank to form the present lake basin due to tectonic movement. The lake sediments accumulated during the last several hundred thousand years. (**b**) An enlarged map of way points (grey rectangle), water temperature inversion points measured by Tantan (red crosses) and vents from 2009 to 2012 (measurement  dates are given in legend). A sediment temperature profile in Fig. 4 was measured at the same point (filled black rectangle) on 27 December 2009. The locations where the images/figures in Figs. 1a–d, 2c,d, 5 and 6 were obtained are indicated by symbols and arrows.

**Figure 4:**
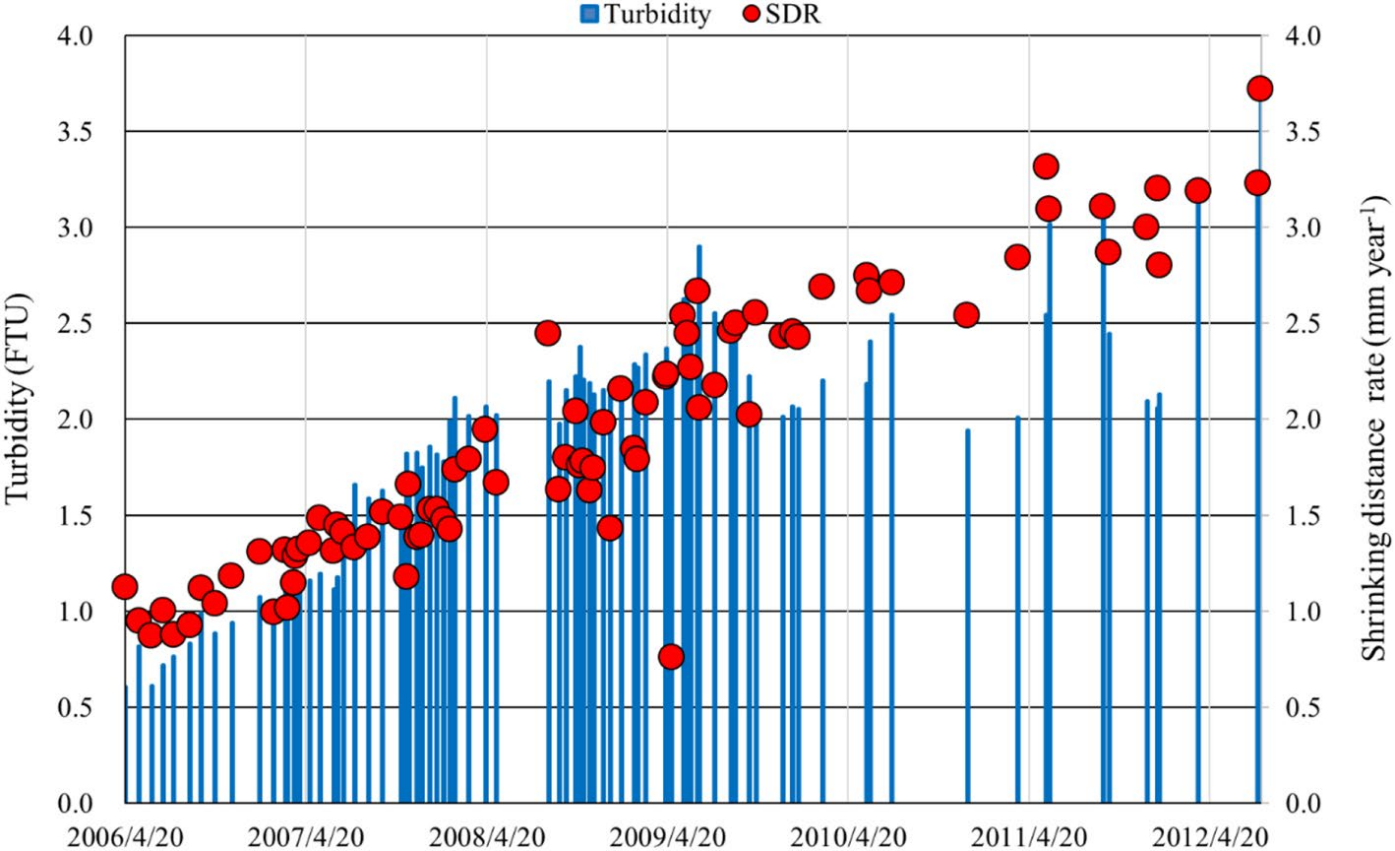
Temporal changes in turbidity (bar graph) at 1 m above the lake bottom and shrinking distance rate (SDR) of Lake Biwa between Hikone (east side) and Kutsuki (west side) from 2006 to 2012 (see Fig. 3) were highly correlated (r^2^ = 0.66 and p < 0.01 for N = 83). Increasing vent number detected by the AUV also corresponded to the increase of lake shrinking distance rate over six years.

Comparing the thickness of accumulated sediments from about 1.5 Ma to the present day^2,33^ with the locations of the benthic vents detected between 2009 and 2012 shows that the vents are located along the area of shallow sedimentation, corresponding roughly to the location of the former mountain range (Fig. 3). A large negative gravity anomaly of -60 mGal was measured around Lake Biwa, indicating a thin crustal layer under the lake or the presence of low-density materials due to faults^34^. This area of the lake bottom not only has many faults but has also been subjected to large earthquakes (> M7.0) every 400–500 years^35^. These background data suggest that the benthic vents found in Lake Biwa did not originate from ground water, but are related to active tectonic movements. For example, using the data from 2006 to 2012, the turbidity [Turb (FTU)] at the deepest point in the lake is positively correlated with the east–west lake shrinking distance rate [SDR (mm year^−1^)] as [Turb] = 0.9866[SDR], where N = 83 and r^2^ = 0.66, which suggests that crustal movements caused the increase in ebullitions (Fig. 4). The M9.1 earthquake off the Pacific coast of Tohoku on 11 March 2011^36–38^ can be considered as one of the typical tectonic events compressing the Japanese Archipelago. Also, various slow earthquakes along the Nankai Trough were detected between 2006 and 2018^39^, and water temperature in several deep wells (< 150 m) along the south coast of the main island in Japan rapidly rose from 2017 to 2019 (c.a. 0.09 K year^−1^, Kamikubo et al*.* personal communication). Benthic vent formation in Lake Biwa was concomitant with these phenomena. Our findings may also be a harbinger of a large earthquake in this area of Japan in the near future.

## Evidence from physical limnology

**Figure 5:**
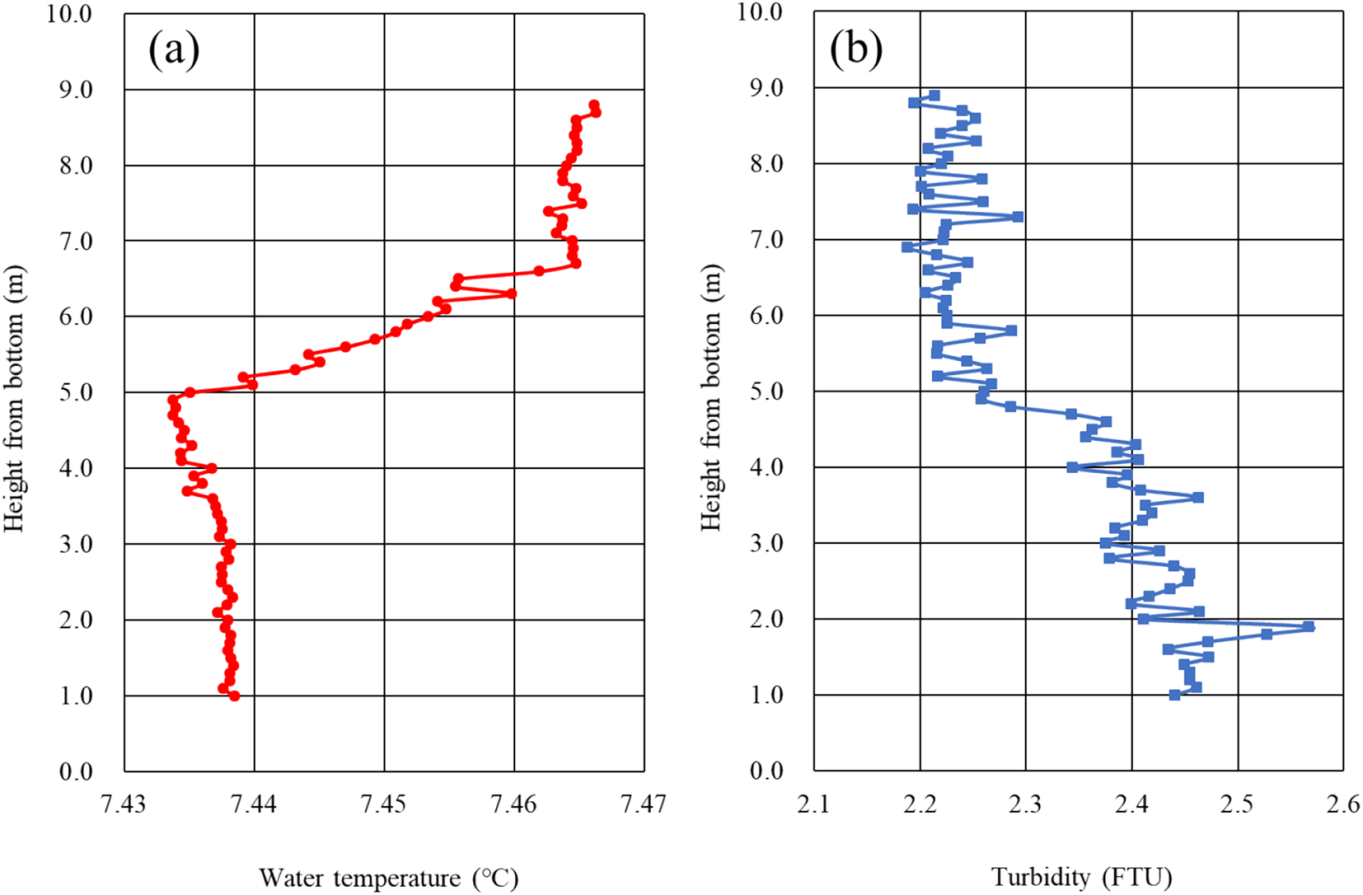
(**a**) Water temperature profile and (**b**) turbidity profile in the benthic boundary layer near the deepest area in the North Basin of Lake Biwa measured on 28 September 2011. Warmer temperature and higher turbidity coexist in the layer from 1 to 5 m above the bottom, which may maintain density stability.

**Figure 6:**
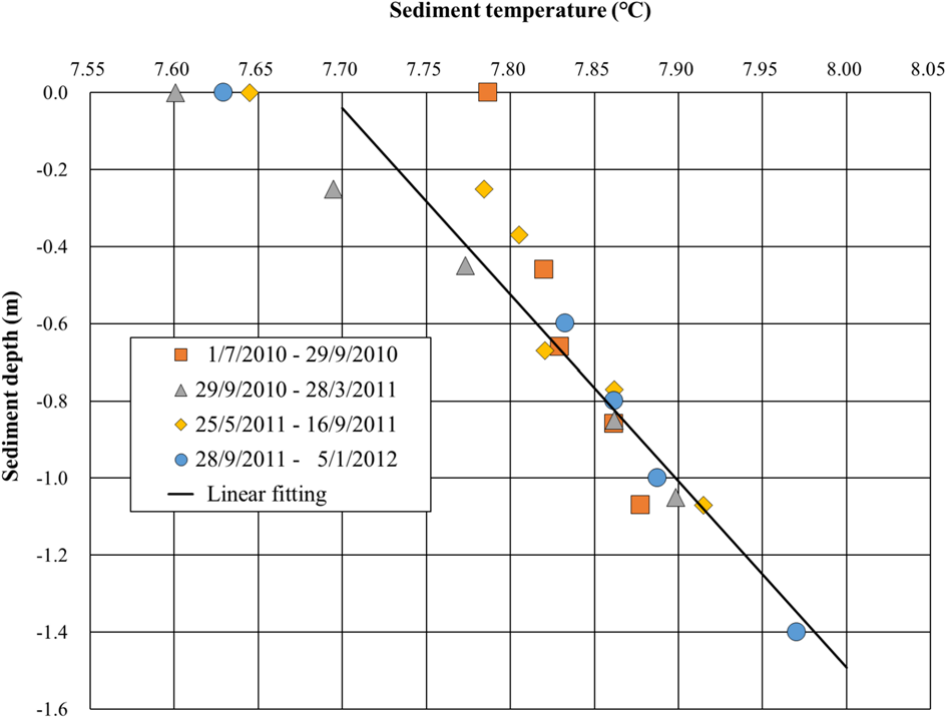
Sediment temperature profiles measured near the deepest point of Lake Biwa from 2010 to 2012 (location indicated in Fig. 3). Plotted are the average data during four periods: July 1 to September 29, 2010; September 29, 2010 to March 28, 2011; May 25 to September 16, 2011; September 28, 2011 to January 5, 2012 and the linear regression fitted line for all data. This line indicates an upward heat flux of 0.2 K m^−1^ from the sediment to the water.

Intensive observations by Tantan were completed along several transects in December 2009, April and December 2010 and January 2012. The water temperature gradient was measured with two thermistors located 0.58 m apart and vertically attached on the head of Tantan. The data showed a temperature inversion, with a maximum gradient of 0.08 K m^−1^ (average 0.003 K m^−1^, upward positive, N = 29,912 and 64.27% > 0) in the benthic boundary near the benthic vents (white smokers in Fig. 1b); i.e., the water temperature near the bottom was slightly but significantly higher than the temperature of the overlying water column. The water temperature inversion points are plotted in Fig. 3. Temperature inversion is usually unstable in freshwater lakes, as mixing of the water column would usually be expected to remove such an inversion. However, if highly turbid water occurs in the benthic boundary layer, the density profile near the bottom can be stable (Fig. 5). Moreover, if the lake bottom is continuously heated, the unstable condition can persist. In order to verify this situation, we deployed 6 RBR thermistors to measure sediment temperature gradients vertically in the bottom sediment near the deepest point in Lake Biwa from 1 July 2010 to 4 January 2012. The results showed a large mean temperature gradient of 0.2 K m^−1^, where the upward gradient was positive (N = 69,455 and SD = 0.014 K m^−1^), indicating strong upward heat flux in the bottom sediments at the deepest point in Lake Biwa (Fig. 3) over the two-year deployment period from 2010 to 2012 (Fig. 6), which was almost 5 times greater than the heat flux outside the benthic vent area (about 0.04 K m^−1^).

Irrespective of the cause for these observed physical changes in the lake bottom, the rapid increase of vents and the associated increased turbidity and GHG releases in Lake Biwa requires systematic ongoing surveillance. The increases in turbidity in the deep benthic layer were not due to benthic shear stress or groundwater inflows, but mainly due to ebullition created by the recently increasing number of vents. These dramatic changes have the potential to not only indicate possible major crustal change but also significantly impact the health of Lake Biwa by increasing influxes to the water column of nutrients, particles and greenhouse gases, and leading to biogeochemical and ecological changes within the lake and ultimately deterioration in lake environmental quality. Indeed, satellite observation of the lake from 2002 to 2018 has indicated a steady increase in chlorophyll concentration, concomitant with algal production (Goto et al. personal communication, 2019). This could significantly impact millions of people that depend upon Lake Biwa for a range of ecosystem services and drinking water, and thus is an environmental problem and priority demanding close monitoring with advanced technology such as an AUV^40^.

## Methods

The world’s first autonomous underwater vehicle (AUV) “Tantan”, developed for lake ecosystem exploration and monitoring, was built in 2000. After test operations in 2000 and 2001, Tantan started to monitor the sediment environments with digital and video cameras in 2002. It has a length of 2 m and weighs 180 kg in air. Maximum speed in water is 1.0 m s^−1^ with a diving depth of 170 m. This automated, untethered vehicle was operated along way points keeping a fixed height of 1 m above lake sediments using an ultra-sonic distance device. The position of Tantan underwater was determined with a SSBL (Super Short Base Line; System Engineering Corp.) and DVL (Doppler Velocity Log; RDI). The AUV drifted about 0.15 km km^−1^ after 123 operations due to mechanical errors. Location coordinates were corrected with a GPS on the water surface at each dive.

A key feature of Tantan is the ability to take two high-definition digital video movies and one digital still picture at the same time^4^. These video cameras faced forward and covered almost the same view. Tantan is also equipped with sensors to observe benthic environmental water temperature, pH, turbidity, dissolved oxygen, conductivity and chlorophyll-a, which were used to determine the environmental conditions in different water bodies during the investigations. Using the two video cameras (SONY HDR-SR12), we obtained video images of vents and were able to identify their position with SSBL (SGK System Giken) and DVL (RDI Doppler Velocity Log). The transect line densities of vents after 2010 in Table 1 were evaluated from linear regression of the accumulated numbers of vents against travel distance.

We used Tantan to measure the vertical water temperature gradient at 1 m above the lake bottom along a transect line with two high resolution temperature probes (RBR with accuracy of 0.002 K). One thermistor was attached to the top foredeck of Tantan, while another was fixed to its bottom surface, and the gradient was calculated over the height difference of 0.58 m between top and bottom probes. The thermistor positions were carefully fixed in order to avoid wrong readings due to AUV battery heating.

We also used a multi-beam echo sounder (MB1, Teledyne, 170 kHz–220 kHz), a quantitative echo sounder (Kaijo, KFC-3000, 70 kHz), and a ROV (NHK original assembly) to identify the positions of vents and gas ebullitions. To identify bottom sediment resuspension, we deployed a 1200 kHz acoustic RDI Doppler current profiler (ADCP) fixed facing downward from 10 m above the bottom at 90 m from September 2010 to January 2012. Gases were collected on two occasions using an original sampling device attached to the ROV, developed by Ritsumeikan University’s robotics team, and analyzed by gas chromatography.

Temperature profiles in bottom sediments were measured with RBR probes located at 0.0, 0.60, 0.80, 1.00, 1.20 and 1.40 m below the water–sediment interface near the deepest point where vents were found. The sampling interval was set to 10 min. The east–west direction shrinking distance rate [SDR (mm year^−1^)] of Lake Biwa was estimated with field data from two stations, Kutsuki (west side) and Hikone (east side), within the dense Global Positioning System (GPS) network in Japan^41^. In Fig. 5, turbidity was measured with a fine-scale profiler (F-probe) at 1 m height above the lake bottom. FTU (Formazin Turbidity Unit) is the most widely used measurement unit for turbidity.

## Data Availability

All data used here are available from the first author.

## References

[1] Horie S (1984). Lake Biwa.

[2] Takemura K, Haraguchi T, Kusumoto S, Itoh Y (2013). Tectonic basin formation in and around Lake Biwa, Central Japan. Mechan. Sedim. Basin Form..

[3] Kumagai M, Vincent WF, Ishikawa K, Aota Y, Kumagai M, Vincent WF (2003). Lessons from Lake Biwa and other Asian lakes. Freshwater Management: Global Versus Local Perspectives.

[4] Kumagai M (2008). Lake Biwa in the context of world lake problems. Verh. Inter. Verein. Limnol..

[5] Kumagai M, Ura T, Kuroda Y, Walker RF (2002). A new autonomous underwater vehicle designed for lake environment monitoring. Adv. Robot..

[6] Oki I (1998). Chromosomes of *Phagocata kawakatsui* and *Bdellocephala annandalei* from Lake Biwa-ko in Honshú, central Japan. Hydrobiologia.

[7] Trevorrow MV, Tanaka Y (1997). Acoustic and in situ measurements of freshwater amphipods (*Jesogammarus annandalei*) in Lake Biwa, Japan. Limnol. Oceanogr..

[8] Ishikawa T, Urabe J (2002). Population dynamics and production of *Jesogammarus annandalei*, an endemic amphipod, in Lake Biwa, Japan. Freshw. Biol..

[9] Svensson J, Leonardson L (1996). Effects of bioturbation by tube-dwelling chironomid larvae on oxygen uptake and denitrification in eutrophic lake sediments. Freshw. Biol..

[10] Crane K, Hecker B, Golubev V (1991). Hydrothermal vents in Lake Baikal. Nature.

[11] Tiercelin J-J (1993). Hydrothermal vents in Lake Tanganyika, East African, Rift system. Geology.

[12] de Ronde CEJ (2002). Discovery of active hydrothermal venting in Lake Taupo, New Zealand. J. Volcanol. Geotherm. Res..

[13] Igarashi G (1992). Mantle helium flux from the bottom of Lake Mashu, Japan. Earth Planet. Sci. Lett..

[14] Nishiura R, Tsunogai U, Ishibashi J, Wakita H, Nojiri Y (1999). Origin of ^13^C-enriched methane in the crater lake Towada, Japan. Geochem. J..

[15] Yamanaka T (2013). Shallow submarine hydrothermal activity with significant contribution of magmatic water producing talc chimneys in the Wakamiko Crater of Kagoshima Bay, southern Kyushu, Japan. J. Volcanol. Geotherm. Res..

[16] Goto N (2019). Evaluation of Organic Carbon Budgets in Lake Biwa for Management of Water Quality and Ecosystem.

[17] Urabe J, Ishida S, Nishimoto M, Weider LJ (2003). *Daphnia pulicaria*, a zooplankton species that suddenly appeared in 1999 in the offshore of Lake Biwa. Limnology.

[18] Deines P, Fink P (2011). The potential of methanotrophic bacteria to compensate for food quantity or food quality limitations in *Daphnia*. Aquat. Microbial Ecol..

[19] Schilder J (2015). The stable carbon isotopic composition of *Daphnia* ephippia in small, temperate lakes reflects in-lake methane availability. Limnol. Oceanogr..

[20] Murase J, Sakai Y, Sugimoto A, Okubo K, Sakamoto M (2003). Sources of dissolved methane in Lake Biwa. Limnology.

[21] Tsunogai U (2020). Dual stable isotope characterization of excess methane in oxic waters of mesotrophic lake. Limnol. Oceanogr..

[22] Tsunogai U (2010). Origin and fate of deep-sea seeping methane bubbles at Kuroshima Knoll, Ryukyu forearc region, Japan. Geochem. J..

[23] Holliday DV, Pieper RE (1980). Volume scattering strengths and zooplankton distributions at acoustic frequencies between 0.5 and 3 MHz. J. Acous. Soc. Am..

[24] Borges AV (2011). Diffusive methane emissions to the atmospherefrom Lake Kivu (Eastern Africa). J. Geophy. Res..

[25] Ostrovsky I, McGinnis DF, Lapidus L, Eckert W (2008). Quantifying gas ebullition with echosounder: the role of methane transport by bubbles in a medium-sized lake. Limnol. Oceanogr. Methods.

[26] Aben RCH, Barros N, van Donk E (2017). Cross continental increase in methane ebullition under climate change. Nat. Commun..

[27] Howarth MJ, Souza AJ (2005). Reynolds stress observations in continental shelf seas. Deep-Sea Res..

[28] Otsubo K, Muraoka K (1988). Critical shear stress of cohesive bottom sediments. J. Hydrol. Eng..

[29] Taniguchi M (1995). Change in groundwater seepage rate into Lake biwa, Japan. Jpn. J. Limnol..

[30] Tsurumaki M, Kobayashi M (1989). Interaction of lake and ground water: Case study of Lake Biwa. J. Geogr..

[31] Kobayashi M (1993). Groundwater seepage into Lake Biwa traced by pollutants. Tracers in Hydrology. IAHS.

[32] Horie S (1983). Paleolimnology of Lake Biwa and the Japanese Pleistocene.

[33] Uemura Y, Taishi H (1990). Active tectonics of the bottom of Lake Biwa and development of its lake basin, southwest Japan. Geogr. Rev. Jpn. B..

[34] Nishida J, Katsura I, Nishimura S (1990). Gravity survey around Lake Biwa, southwest Japan. J. Phys. Earth..

[35] Goto S, Yamanom M, Kinoshita M (2005). Thermal response of sediment with vertical fluid flow to periodic temperature variation at the surface. J. Geophy. Res..

[36] Geller G (2011). Shake-up time for Japanese seismology. Nature.

[37] Sato M (2011). Displacement above the hypocenter of the 2011 Tohoku-Oki earthquake. Science.

[38] Ozawa S (2011). Coseismic and postseismic slip of the 2011 magnitude-9 Tohoku-Oki earthquake. Nature.

[39] Yokota Y, Ishikawa T (2020). Shallow slow slip events along the Nankai Trough detected by GNSS-A. Sci. Adv..

[40] Clarke T (2003). Robots in the deep. Nature.

[41] Sagiya T, Miyazaki S, Tada T (2000). Continuous GPS array and present-day crustal deformation of Japan. Pure Appl. Geophys. J. Int..

